# Leveraging AutoML to optimize dataset selection for improved breast cancer variants pathogenicity prediction

**DOI:** 10.1016/j.csbj.2025.10.052

**Published:** 2025-10-27

**Authors:** Rahaf M. Ahmad, Noura AlDhaheri, Mohd Saberi Mohamad, Bassam R. Ali

**Affiliations:** a Department of Genetics and Genomics, College of Medicine and Health Sciences, United Arab Emirates University, United Arab Emirates; b Centre for Advanced Analytics, CoE for Artificial Intelligence, Faculty of Engineering & Technology, Multimedia University, Melaka 75450, Malaysia; c Department of Biosystems Engineering, Faculty of Agricultural Technology, Universitas Brawijaya, Malang, East Java, Indonesia; d Institute For Data Innovation and Artificial Intelligence, Cranbourne East 3977, Victoria, Australia

**Keywords:** Breast cancer, Pathogenicity prediction, Automated machine learning, Dataset optimization, Genetic variants

## Abstract

Breast cancer (BC) remains one of the most prevalent and lethal malignancies worldwide, with its onset shaped by complex interactions between germline predispositions, environmental exposures, and accumulated somatic mutations. Accurate prediction of variant pathogenicity is essential for identifying high-risk individuals, guiding early detection, and tailoring treatment strategies. However, existing computational tools often lack disease-specific training and fail to generalize across diverse variant datasets. To address this gap, we systematically benchmarked the predictive utility of four distinct variant datasets using three Automated Machine Learning (AutoML) frameworks-TPOT, H2O AutoML, and MLJAR. Our goal was to evaluate how dataset composition influences classification performance and to identify the optimal dataset for BC-specific pathogenicity prediction. Among the datasets evaluated, Dataset-2-curated from both cancer-specific and non-cancer databases, consistently yielded the highest predictive performance across all frameworks. H2O AutoML achieved a peak accuracy of 99.99 %, while TPOT and MLJAR also exhibited robust generalization on this dataset. Feature importance analyses revealed strong convergence across frameworks, highlighting conservation scores and pathogenicity metrics as dominant predictors. Interpretability techniques including SHAP, permutation importance, and LIME further validated the biological relevance and transparency of the models. This study presents a scalable, interpretable AutoML benchmarking framework tailored to the clinical prioritization of BC variants. By demonstrating the superiority of cancer-specific, disease-relevant datasets, our findings underscore the critical importance of thoughtful dataset design in machine learning pipelines for genomic medicine. Beyond BC, this framework is readily transferable to other genetic disorders, providing a foundational tool for precision diagnostics and the advancement of personalized oncology.

## Highlights

• AutoML benchmarking identifies optimal dataset for breast cancer variant prediction.
• Cancer-specific datasets yield higher predictive performance than general datasets.
• SHAP, LIME, and permutation importance confirm model interpretability.
• Workflow supports scalable, transferable precision oncology applications.

## Graphical Abstract

**Figure:**
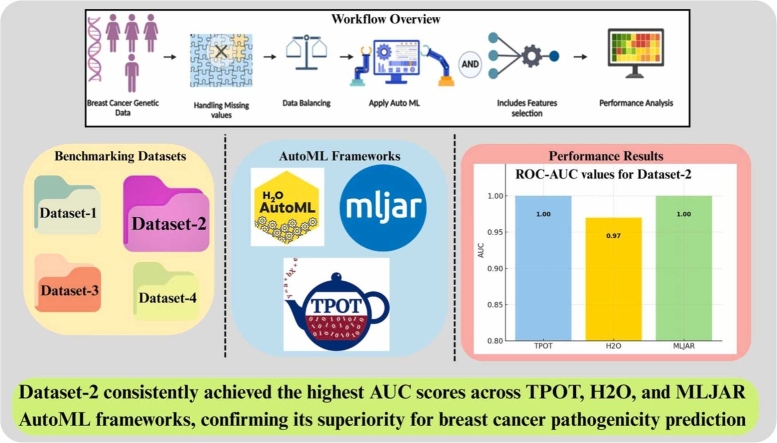

## 1 Background

Cancer is characterized by the unregulated expansion and multiplication of cancer cells, resulting in an abnormal increase in both cell size and number. Although it can originate in any organ, the specific name of the cancer reflects its location at onset-for instance, cancers arising in breast tissue are classified as breast cancer (BC) [1]. Globally, BC poses a major public health concern, with millions of women affected each year [2]. According to the World Health Organization (WHO), around 1.5 million new cases are diagnosed annually globally, contributing to high morbidity and mortality rates. In 2022, the WHO reported that 2.3 million women worldwide were diagnosed with BC, resulting in 670,000 deaths [3]. This condition stems from various genetic, environmental, and lifestyle factors, including inherited and acquired mutations in the *BRCA1*, *BRCA2* and many other genes [4]. Despite substantial research progress, many aspects of the genetic and molecular factors driving BC remain insufficiently understood. Determining genetic predisposition risk and early detection are crucial for improving treatment outcomes and survival rates. However, the massive influx of genetic data from multiple initiatives employing modern high-throughput DNA sequencing technologies makes the classification of variants quite challenging, highlighting the urgent necessity for advanced and robust approaches.

Automated Machine Learning (AutoML) is an evolving discipline that focuses on streamlining the development of machine learning models by reducing manual intervention. The primary objective of AutoML is to enhance efficiency and productivity by automating various labor-intensive aspects of the machine learning pipeline. For years, researchers have explored methodologies to generate high-quality predictive models while minimizing the need for extensive manual configuration, spanning from data preprocessing to algorithm selection and hyperparameter tuning. AutoML has shown significant potential in optimizing and selecting machine learning models for BC diagnosis [5]. Automated Machine Learning (AutoML) frameworks offer significant advantages over traditional machine learning methods by automating critical tasks like data preprocessing, selecting features, tuning hyperparameters, and evaluating models. Unlike traditional ML, which requires extensive domain expertise and manual intervention, AutoML streamlines these processes, reducing human error and improving reproducibility, particularly in biomedical research where datasets are often large and complex [6].

TPOT is an open-source software tool built on the scikit-learn library, leverages genetic programming to iteratively refine machine learning pipelines, ensuring optimal model performance through a process inspired by natural selection. It excels in creating diverse, high-performing pipelines tailored to dataset characteristics, particularly in tasks involving regression and classification with fewer than 50 classes, making it highly efficient and computationally effective [7], [8], [9].

On the other hand, H2O is another open-source platform designed for scalability, integrating in-memory distributed computing to handle large datasets efficiently. Supporting multiple programming languages, including Python and R, and providing extensive functionality through its AutoML module, H2O employs robust ensemble methods like Distributed Random Forests to rank high-performing models and streamline predictive analytics [5].

Additionally, MLJAR AutoML has emerged as a powerful, user-friendly, and fully automated machine learning framework designed to facilitate rapid model development and interpretation, particularly in biomedical and healthcare domains. It encapsulates the entire machine learning pipeline-including preprocessing, feature engineering, model selection, and tuning-into a single, streamlined process. Unlike many traditional frameworks, MLJAR offers built-in interpretability by generating comprehensive, human-readable HTML reports that include learning curves, confusion matrices, and feature importance scores [10]. This makes MLJAR particularly well-suited for genetic and clinical research, where transparency and reproducibility are essential. It supports a wide array of algorithms such as LightGBM, XGBoost, CatBoost, and neural networks, and includes advanced ensemble strategies like stacking and blending. Additionally, its flexible experimentation modes (Explain, Perform, and Compete) allow for fast benchmarking and scalable experimentation, making it an effective solution for analyzing large, heterogeneous genetic datasets. Although MLJAR has not yet been widely applied to BC variant classification, it has shown strong performance in similar biomedical contexts, including tumor classification and disease risk prediction, underscoring its potential utility in genetic pathogenicity modeling [10].

Together, these frameworks complement each other, TPOT’s evolutionary pipeline optimization aligns well with H2O’s scalability and robust ensemble capabilities, while MLJAR offers ease of use, integrated interpretability, and reproducibility, creating a powerful system for evaluating BC pathogenicity prediction datasets. TPOT has been utilized to optimize machine learning algorithms for pathogenicity prediction, demonstrating its efficacy in identifying optimal models when applied to training datasets [11]. While H2O was not directly applied for the pathogenicity prediction of BC, it outperformed other machine learning algorithms in predicting estrogen receptor status using BC metabolomics data [12]. Beyond that, both frameworks have demonstrated their versatility across biomedical applications, with TPOT identifying key feature combinations in metabolomics and transcriptomics and H2O excelling in risk prediction for conditions like cervical and BC, as well as structural analyses of genetic variants [13], [14]. By eliminating the need for extensive domain expertise and handling the complexities of large, heterogeneous datasets, AutoML frameworks significantly accelerate predictive model discovery and improve reproducibility, making them invaluable for precision medicine and genetic research.

Several open-source AutoML frameworks have gained prominence for their effectiveness in automating these processes [15]. However, AutoML frameworks were not previously used to benchmark BC variants datasets for pathogenicity prediction. Therefore, the lack of dataset benchmarking studies leaves the question of “which dataset is optimal for developing a pathogenicity prediction tool?” unanswered. Thus, this study aims to employ AutoML frameworks to benchmark and identify the optimal dataset that can be used in further development of a pathogenicity prediction tool tailored for BC. This outcome will pave the road towards early detection of BC in addition to the possibility of personalizing treatments for each patient based on the causative variants. By evaluating four distinct datasets based on size, quality, and source, this work fills a critical gap in BC research, representing the first effort to benchmark BC-specific genetic variant datasets to determine the optimal composition for accurate pathogenicity prediction.

## 2 Methods

### 2.1 Data collection and preprocessing

Genetic variants were downloaded from several online databases, some of them were cancer specific databases including COSMIC [16] (https://cancer.sanger.ac.uk/cosmic) and CBioPortal [17] (https://www.cbioportal.org/datasets), while others were general databases such as ClinVar [18] (https://www.ncbi.nlm.nih.gov/clinvar/) and HGMD Professional 2024.3 [19] (https://www.hgmd.cf.ac.uk/). Yet, all variants collected were filtered to include cancer related phenotypes. Next, the variants were annotated using the Variant Effect Predictor (VEP) from Ensembl (https://asia.ensembl.org/info/docs/tools/vep/index.html) [20], and the available data was thoroughly reviewed. After careful examination, the data was categorized into two main sets: variants downloaded from cancer-specific databases and those obtained from both cancer-specific and general databases. To ensure reproducibility despite the inclusion of HGMD Professional, all analyses can be replicated using publicly available subsets from ClinVar, COSMIC, BRCAExchange, and cBioPortal as long as the dataset structure matches the structure of the sample file uploaded on the Github repository. Further, each set was divided into 2 datasets, ending up with 4 datasets. All datasets were balanced by adding benign variants for the corresponding genes from the GnomAD database. The first dataset (Dataset-1) included variants from the ClinVar and the HGMD pro v2024.3 databases, those databases are not considered to be specific for cancers, therefore we considered dataset-1 a general dataset. Dataset-1 originally included 1428 Pathogenic variants and 932 Benign variants, the final version of Dataset-1 that was used to generate the results of this manuscript included 1428 Pathogenic variants and 1428 Benign variants.

The second dataset (Dataset-2) included variants from COSMIC [16], CBioPortal [17], BRCAExchange [21] (https://brcaexchange.org/) and TCGA [22] (https://www.cancer.gov/ccg/research/genome-sequencing/tcga) databases which are cancer specific databases, in addition to ClinVar [18] and HGMD [19] to increase the volume of the training data. Dataset-2 originally included 3721 Pathogenic variants and 2889 Benign variants. The final version of Dataset-2 that was used to generate the results below included 3721 Pathogenic variants and 3721 Benign variants. The first two datasets were filtered to include the variants related to BC only, this was done by filtering out any gene that is not in our gene lists highlighted by previous work [23], [24]. The final list of genes present in Dataset-2 is summarized in Table 1 below. While the next two datasets were not filtered, and kept as collected based on the phenotype which included any cancer related phenotype variants for all cancer types like, breast, renal, lung, brain, skin, etc.

**Table 1:** List of genes in dataset-2.

**Genes**					
**TP53**	**BRIP1**	**NRAS**	**SMARCA4**	**PDGFRA**	**RAD51**
**BRCA2**	**FANCM**	**PARP2**	**APC**	**WRAP53**	**SF3B1**
**BRCA1**	**FBXW7**	**POLD1**	**GNAS**	**CASP8**	**CDKN2A**
**ERBB2**	**NBN**	**RAD51D**	**STK11**	**SDHA**	**CPS1**
**ATM**	**RB1**	**SETBP1**	**HRAS**	**ATXN7**	**DIS3L2**
**PTEN**	**ERBB3**	**ARPC1B**	**RAD51B**	**CACNA2D3**	**EIF2B5**
**CDH1**	**RINT1**	**GEN1**	**MET**	**DCLRE1B**	**ERCC5**
**MSH6**	**SLX4**	**PTPRF**	**PMS2**	**FAM175A**	**FGFR3**
**MSH2**	**RET**	**SYNE2**	**EGFR**	**MTOR**	**GPC3**
**CHEK2**	**IDH1**	**ALCAM**	**RAD50**	**PPP2R1A**	**JAK2**
**CTNNA1**	**MUTYH**	**ALK**	**CDK12**	**SMAD4**	**KIT**
**PIK3CA**	**NTHL1**	**CDC73**	**MAP3K1**	**SUFU**	**MRE11A**
**NF1**	**PPM1D**	**FANCI**	**TSC2**	**WRN**	**MYO7A**
**PMS1**	**RHOA**	**FGFR4**	**RAD54L**	**GALNT12**	**PTPN11**
**MLH1**	**BMPR1A**	**RECQL**	**BLM**	**EPCAM**	**AR**
**BARD1**	**KRAS**	**SHBG**	**FANCD2**	**PALLD**	**FANCC**
**PALB2**	**POLE**	**VHL**	**RAD51C**	**NF2**	**FLNC**
**ESR1**	**CDKN1A**	**MED12**			

The third dataset (Dataset-3) included variants from the ClinVar [18] and HGMD databases [19], those databases are not considered to be specific for cancers, therefore similar to Dataset-1, we considered Dataset-3 the second general dataset. Dataset-3 originally included 5107 Pathogenic variants and 3652 Benign variants, the final version of Dataset-3 that was used to generate the results below included 5107 Pathogenic variants and 5107 Benign variants.

The fourth dataset (Dataset-4) included variants from COSMIC [16], CBioPortal [17], BRCAExchange [21] and TCGA [22] databases which are cancer specific databases, in addition to ClinVar [18] and HGMD [19]. Dataset-4 originally included 16179 Pathogenic variants and 9132 Benign variants, the final version of Dataset-4 that was used to generate the results below included 16179 Pathogenic variants and 16179 Benign variants.

The next step was data preprocessing, where missing values were imputed through the KNNImputer package, and categorical variables were label-encoded through the label encoder package. KNN imputation was used to handle missing values due to its ability to estimate missing data based on the similarity of neighboring data points, ensuring minimal distortion in the dataset [25]. All datasets were balanced by adding benign variants for the corresponding genes from the GnomAD database, thereby enhancing the model's ability to learn from underrepresented classes and to avoid any bias during splitting, training, or any other computational task and to make sure all datasets are in the same structure before applying the AutoML tools. To minimize potential bias introduced by benign variants from gnomAD, benign and pathogenic variants were matched by gene and variant type to preserve comparable allele-frequency distributions across populations. Benign variants were expanded based on ACMG/AMP population frequency criteria, excluding those labeled pathogenic in ClinVar. Gene-level balancing ensured proportional representation of variants from major cancer-associated genes such as *BRCA1*, *BRCA2*, and *TP53*. Cross-validation was stratified jointly by gene and class, ensuring fair evaluation and preserving biological relevance across all models. All datasets underwent the same preprocessing and it was done using Python 3.10 using the Pycharm IDE and was done identically through all datasets.

### 2.2 AutoML workflow

To optimize the predictive performance of multiple BC variant datasets, we employed a two-phase AutoML workflow using three open-source frameworks: TPOT, H2O AutoML, and MLJAR AutoML. The overarching goal was to determine the most suitable dataset for pathogenicity prediction by comparing model accuracy, stability, and interpretability across frameworks. The workflow was structured into two phases: the first identified the best-performing random seed for each dataset based on AUC, and the second used these seeds for full model training and evaluation.

In Phase 1, each dataset was pre-processed by removing non-informative columns and encoding the clinical significance target label. A stratified 80:20 train-test split was applied, and each dataset was evaluated across five random seeds (42, 101, 202, 303, and 404) to ensure robustness and reproducibility.

Each AutoML framework was independently applied across all seeds. TPOT and MLJAR performed internal cross-validation during pipeline optimization, while H2O used its leaderboard mechanism to rank models. The primary performance metric was the area under the ROC curve (AUC) calculated on the test set. Seed-wise AUC values were visualized, and the top-performing seed for each dataset was selected for Phase 2.

In Phase 2, each dataset was re-evaluated using its corresponding best-performing seed. Prior to model training, statistical feature selection was performed to enhance model performance, reduce overfitting, and improve interpretability. Specifically, features were ranked using both F-test p-values (ANOVA) and the Kruskal–Wallis test, enabling robust identification of relevant features under both parametric and non-parametric assumptions. Using both tests in parallel allows us to capture a broader spectrum of significant features, improving the stability and generalizability of model training by mitigating bias introduced by distributional assumptions [26], [27]. The top-ranked features were then selected and used to reduce the dimensionality of each dataset before model training. Feature selection was guided by both ANOVA (F-test) and Kruskal–Wallis (H-test), retaining variables with p < 0.05 in both analyses. In total, 20 features met this criterion and were used to train all four datasets to maintain consistency. The top-ranked variables included ada_score, rf_score, MaxEntScan_diff, MaxEntScan_alt, Enformer_SAR, DEOGEN2_score, Enformer_SAD, VARITY_R_score, MutPred_score, and VARITY_R_LOO_score, reflecting a strong contribution from ensemble and deep-learning-based predictors.

Final models from TPOT, H2O, and MLJAR were retrained using these statistically optimized feature subsets and evaluated using a comprehensive suite of metrics: AUC, precision, recall, F1-score, Cohen’s kappa, and Matthews correlation coefficient (MCC). To ensure statistical robustness, all results were based on 5-fold cross-validation, and 95 % bootstrapped confidence intervals (n = 1000) were computed for each metric. This combination of statistical filtering and automated modelling provided a reproducible and interpretable approach to identifying the most predictive features for BC variant pathogenicity.

To facilitate visual comparison, combined ROC curves were generated across frameworks and datasets. Feature importance was computed using permutation importance for TPOT, built-in variable importance for H2O, and MLJAR’s native explainability reports. The top 10 contributing features per dataset were visualized to support transparent interpretation and downstream clinical relevance.

**Fig. 1:**
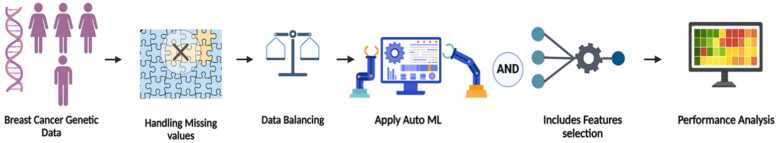
The workflow of the employed experimental methodology.

The entire workflow was implemented in Python using libraries such as scikit-learn, TPOT, H2O, and matplotlib. All experiments were conducted on Google Colab Pro with GPU support, ensuring efficient execution while maintaining reproducibility. A schematic overview of the complete AutoML workflow is presented in Fig. 1.

## 3 Results

To evaluate the most suitable dataset for pathogenicity prediction of BC variants, we first conducted a seed-wise benchmarking across five random seeds (42, 101, 202, 303, and 404) using TPOT, H2O AutoML, and MLJAR AutoML frameworks. For each dataset, the seed that yielded the highest test AUC was selected as the optimal seed for further evaluation. To minimize within-gene correlation, stratified data splits using StratifiedKFold was applied so that class balance (pathogenic vs. benign) was maintained in both training and test subsets. Although the split was performed at the variant level, group-based checks ensured that high-frequency genes such as *BRCA1*, *BRCA2*, and *TP53* were proportionally represented, preventing bias from overrepresented loci. This initial phase ensured that final comparisons were conducted under the most favorable conditions for each dataset and framework. The selected seeds were seed 303 for Dataset-1 (H2O), seed 202 for Dataset-1 (TPOT and MLJAR), seed 101 for Dataset-2 (H2O), seed 404 for Dataset-2 (TPOT and MLJAR) and seed 42 for Dataset-3 (MLJAR). Seed 404 yielded the best performance across all three frameworks for Dataset-4 and Dataset-3 (H2O and TPOT). These choices were based on the AUC values reported in the multi-seed comparison Table 2 below. Interestingly, Dataset-2 consistently yielded the highest AUC values across all frameworks, followed by Dataset-4 and Dataset-3. Dataset-1 had the lowest performance due to limited specificity and size.

**Table 2:** AUC scores across five random seeds (42, 101, 202, 303, and 404) for each AutoML framework (H2O, MLJAR, TPOT) and dataset. The best-performing seed for each combination is bolded and was selected for full model training and final evaluation.

**Dataset-1**														
**H2O**					**MLJAR**					**TPOT**				
**42**	**101**	**202**	**303**	**404**	**42**	**101**	**202**	**303**	**404**	**42**	**101**	**202**	**303**	**404**
0.950645	0.971974	0.976687	**0.990823**	0.96255	0.856895	0.932788	**0.9437**	0.93006	0.895585	0.90625	0.964286	**0.97619**	0.968006	0.96131
**Dataset−2**														
**H2O**					**MLJAR**					**TPOT**				
**42**	**101**	**202**	**303**	**404**	**42**	**101**	**202**	**303**	**404**	**42**	**101**	**202**	**303**	**404**
0.998606	**0.999946**	0.999945	0.99978	0.999916	0.967605	0.972648	0.967412	0.968464	**0.972819**	0.981051	0.999991	0.997721	0.99983	**0.999996**
**Dataset−3**														
**H2O**					**MLJAR**					**TPOT**				
**42**	**101**	**202**	**303**	**404**	**42**	**101**	**202**	**303**	**404**	**42**	**101**	**202**	**303**	**404**
0.994191	0.988354	0.991206	0.99248	**0.994295**	**0.926946**	0.910489	0.918088	0.912635	0.918618	0.993727	0.995627	0.989451	0.942749	**0.99766**
**Dataset−4**														
**H2O**					**MLJAR**					**TPOT**				
**42**	**101**	**202**	**303**	**404**	**42**	**101**	**202**	**303**	**404**	**42**	**101**	**202**	**303**	**404**
0.99643	0.996788	0.995028	0.99676	**0.997223**	0.899175	0.877493	0.899531	0.885767	**0.905752**	0.99499	0.850189	0.996506	0.98377	**0.999034**

Note: Bold values are the highest values representing the top performing metrics.

Following seed optimization, the final models were trained on the corresponding best seed splits and evaluated using a comprehensive set of metrics, including AUC, precision, recall, F1-score, Cohen’s kappa, and MCC, along with 95 % confidence intervals. Across all frameworks, Dataset-2 consistently emerged as the best-performing dataset. For example, H2O achieved an AUC of 1.00 [1.00, 1.00], while TPOT and MLJAR reported 0.996 [0.995, 0.997] and 0.973 [0.965, 0.980], respectively. This superior performance can be attributed to the dataset’s specificity to BC, as it was derived from curated sources such as BRCAExchange and COSMIC, and its relatively large size compared to Dataset-1. In contrast, Dataset-4, although sizable, contained variants from multiple cancer types, which may have introduced additional noise and slightly reduced its performance.

**Fig. 2:**
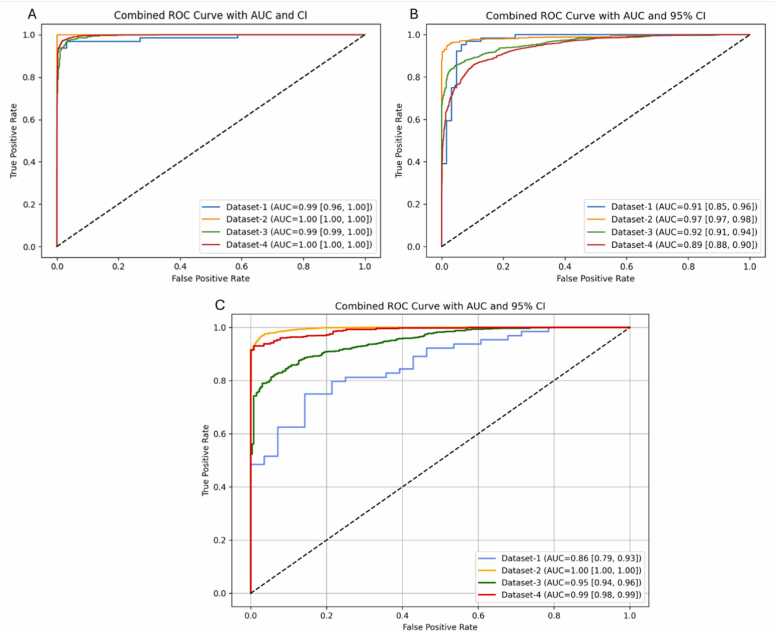
Combined ROC-AUC curves with 95 % confidence intervals for all four datasets across the three AutoML frameworks: (A) H2O AutoML, (B) MLJAR AutoML, and (C) TPOT AutoML.

The combined ROC-AUC curves for the best-performing models from each framework across all datasets are shown in Fig. 2. Fig. 2A illustrates nearly perfect performance of H2O AutoML for Dataset-2 and Dataset-4, both achieving an AUC of 1.00 [1.00, 1.00], while Dataset-1 lags slightly with an AUC of 0.99 [0.96, 1.00]. Fig. 2B shows that for MLJAR AutoML, Dataset-2 again leading (AUC = 0.97 [0.97, 0.98]), followed by Dataset-3 and Dataset-1, with Dataset-4 exhibiting lower sensitivity at certain thresholds (AUC = 0.89 [0.88, 0.90]). In contrast, Fig. 2C confirms Dataset-2’s superiority for TPOT AutoML with an AUC of 1.00 [1.00, 1.00], while Dataset-1 underperforms substantially with an AUC of 0.86 [0.79, 0.93]. These curves consistently demonstrate the robustness of Dataset-2 across all frameworks, reinforcing its biological relevance and optimal composition for BC pathogenicity prediction.

**Fig. 3:**
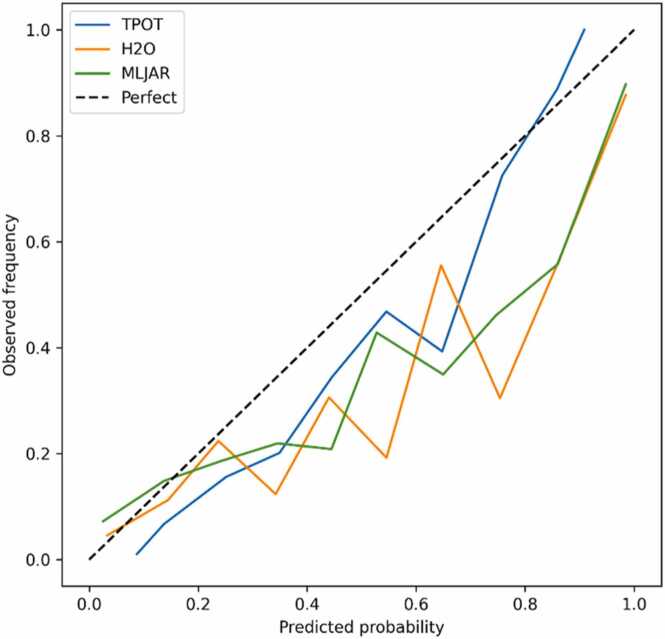
Calibration curves for Dataset-2 across AutoML frameworks (TPOT, H2O, and MLJAR).

These findings are also evident in Table 3, which summarize all evaluation metrics across frameworks. Notably, Dataset-1 showed the weakest performance, likely due to its limited sample size and use of non-cancer-specific annotations. To further assess reliability beyond discrimination, calibration analysis was performed for Dataset-2, the best-performing dataset across all frameworks. The calibration curve in Fig. 3 compare predicted probabilities against observed outcome frequencies. H2O and MLJAR predictions showed near-ideal calibration across the probability range, while TPOT slightly overestimated probabilities for high-confidence predictions. Overall, the results confirm that the models not only achieve high AUC values but also produce clinically interpretable, well-calibrated probability outputs.

**Table 3:** Summary of the performance metrics for all datasets against all three AutoML frameworks with the lower and upper confidence interval for each metric.

**Dataset**	**Dataset-1**			**Dataset-2**			**Dataset-3**			**Dataset-4**		
**Framework**	**H2O**	**MLJAR**	**TPOT**	**H2O**	**MLJAR**	**TPOT**	**H2O**	**MLJAR**	**TPOT**	**H2O**	**MLJAR**	**TPOT**
**Seed**	**303**	**202**	**202**	**101**	**404**	**404**	**404**	**42**	**404**	**404**	**404**	**404**
**AUC**	0.985615079	0.912946429	0.861049107	**0.999965669**	**0.973393219**	**0.996033945**	0.994601624	0.923653185	0.947773504	0.996960569	0.892962945	0.988649997
**AUC CI Lower**	0.960864953	0.851202326	0.787290703	0.999880452	0.96570205	0.995045561	0.992471234	0.910534974	0.93530617	0.996095856	0.884887192	0.983251994
**AUC CI Upper**	1	0.959305716	0.927518915	1	0.980108836	0.996911773	0.996505064	0.935082665	0.958982206	0.997752692	0.900507464	0.992821228
**Precision**	**1**	0.896551724	0.866666667	**1**	**0.998567335**	**0.98003743**	0.975806452	0.945041816	0.910028116	0.979744469	0.867581602	0.970731707
**Precision CI Lower**	1	0.811320755	0.777777778	1	0.995588235	0.974869584	0.965051207	0.928117561	0.892944373	0.974820543	0.853993842	0.958423907
**Precision CI Upper**	1	0.967226335	0.951612903	1	1	0.984510724	0.984360255	0.960141988	0.926677254	0.984479783	0.880576942	0.980792893
**Recall**	0.890625	0.8125	0.8125	**0.99023199**	**0.851037851**	**0.970951792**	0.947162427	0.774730656	0.950097847	0.971569839	0.722805933	**0.971916972**
**Recall CI Lower**	0.806439576	0.714182195	0.714285714	0.982455112	0.826346349	0.964829224	0.932549976	0.747227175	0.935667799	0.965472243	0.705040585	0.959752403
**Recall CI Upper**	0.961590909	0.904109589	0.903241315	0.996350476	0.874695136	0.976404955	0.960880306	0.800014521	0.963512146	0.977233321	0.737512268	0.982001615

Note: Bold values are the highest values representing the top performing metrics.

**Fig. 4:**
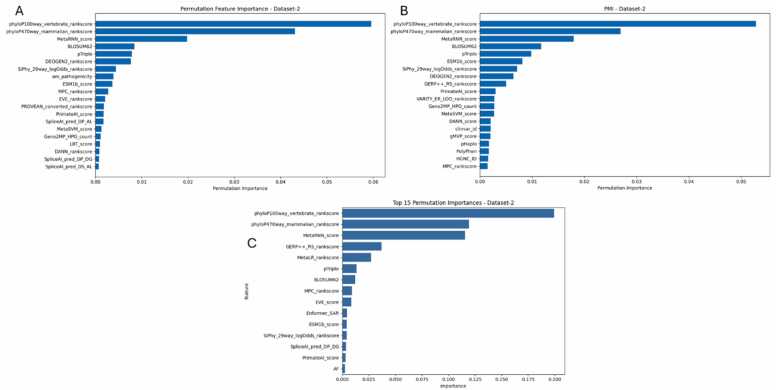
Permutation feature importance for Dataset-2 across AutoML frameworks: (A) H2O, (B) MLJAR, and (C) TPOT. All frameworks consistently rank conservation-based features (phyloP100way, phyloP470way) and ensemble meta-predictors (e.g., MetaRNN, pTriplo, DEOGEN2) among the top contributors to model output.

To interpret which features contributed most to the models’ predictions, we applied permutation feature importance on Dataset-2 across all three frameworks. As shown in Fig. 4, despite architectural differences, all frameworks highlighted a consistent subset of influential features. Fig. 4A showed phyloP100way and phyloP470way conservation scores, along with MetaRNN_score, as the top-ranked contributors, reflecting the importance of evolutionary conservation in variant interpretation for the H2O framework. Fig. 4B revealed a similar trend, with phyloP and MetaRNN features dominating the rankings, followed by diverse ensemble meta-predictors such as PrimateAI, SiPhy, and GERP+ + for the MLJAR framework. Fig. 4C emphasized conservation and ensemble features for TPOT framework but also included MetaLR, MPC, and deep learning scores like Enformer_SAR, indicating TPOT’s flexibility in pipeline composition and feature interaction discovery. These consistent rankings affirm the biological relevance of conservation- and ensemble-based features in BC pathogenicity prediction. Moreover, framework-specific variations-such as TPOT’s inclusion of deep learning-derived scores-highlight how different AutoML approaches prioritize features depending on model complexity and optimization strategy.

**Fig. 5:**
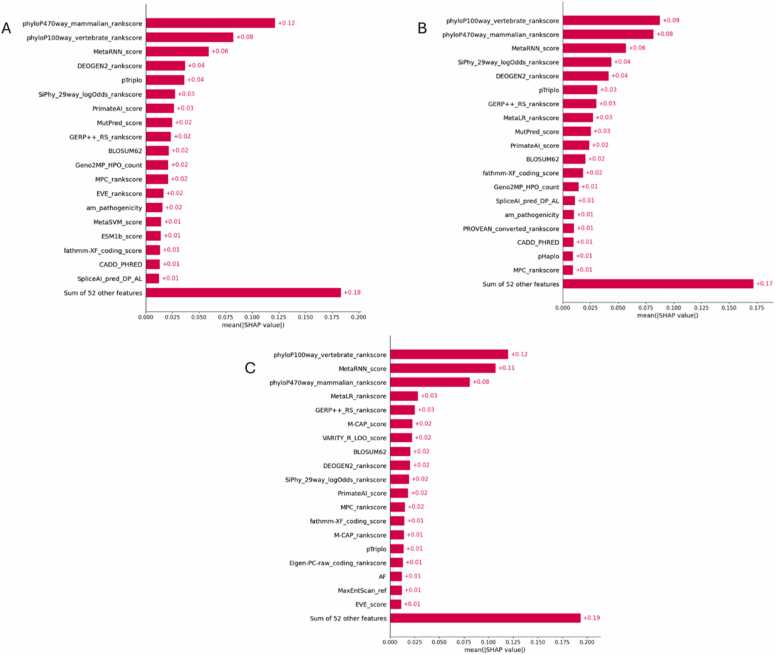
SHAP means absolute bar plot for Dataset-2 across AutoML frameworks: (A) H2O, (B) MLJAR, and (C) TPOT.

To complement permutation importance and gain insight into both global and local model behavior, SHAP (SHapley Additive exPlanations) values were computed for Dataset-2 across all AutoML frameworks. The SHAP bar plot for H2O AutoML on Dataset-2 shown in Fig. 5A reveals that phyloP470way_mammalian_rankscore, phyloP100way_vertebrate_rankscore, and MetaRNN_score were the most influential features, contributing the highest average SHAP values. These top-ranked features reflect the importance of conservation scores and ensemble pathogenicity predictions in determining variant pathogenicity. The compact feature profile indicates H2O’s focus on a small, high-impact subset of features, supporting the stability of its predictive performance. Fig. 5B of the MLJAR AutoML’s SHAP bar plot highlights a slightly broader feature landscape. While phyloP100way, phyloP470way, and MetaRNN_score remain dominant, other predictors such as SiPhy_29way_logOdds_rankscore, MetaLR, and MutPred_score also make significant contributions. This suggests that MLJAR balances a concise core of highly predictive features with additional supporting features that enhance its interpretability and model robustness. TPOT AutoML demonstrates a wider spread of contributing features in its SHAP bar plot as shown in Fig. 5C. Alongside phyloP scores and MetaRNN, TPOT ranks features such as GERP+ +, MetaLR, and even MaxEntScan_ref, indicating a diverse use of conservation, functional annotation, and deep learning-derived features. This reflects TPOT’s flexible pipeline design, which leverages a richer combination of signals to optimize performance.

**Fig. 6:**
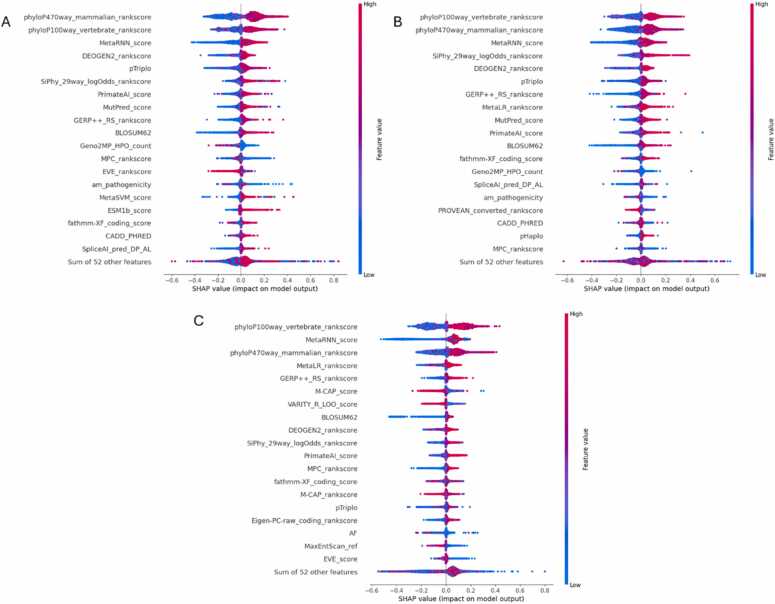
SHAP beeswarm plot for Dataset-2 across AutoML frameworks: (A) H2O, (B) MLJAR, and (C) TPOT.

The beeswarm plot for H2O AutoML in Fig. 6A shows a consistent shift of high-conservation and ensemble score features toward pathogenic predictions. For instance, high values of phyloP470way and MetaRNN strongly push predictions rightward, indicating pathogenicity. The clustering pattern reflects H2O’s tendency to rely on a stable feature set that offers consistent directional impact. Fig. 6B of the MLJAR’s beeswarm plot presents a wider distribution of SHAP values, with key features like phyloP100way, MetaRNN, and SiPhy influencing predictions variably based on input ranges. This variation shows MLJAR’s sensitivity to subtle shifts in feature values, reflecting a more nuanced interpretability landscape. In TPOT’s beeswarm visualization shown in Fig. 6C, features such as phyloP, MetaRNN, MetaLR, and GERP+ + have strong directional impacts, while AF and MaxEntScan_ref show moderate influence. The broader spread across features and directions is consistent with TPOT’s diverse and data-driven pipeline construction, offering insights into both common and context-specific decision paths. To further contextualize these top-ranked features, Table 4 summarizes their biological relevance and pathway-level associations derived from KEGG and Reactome annotations.

**Table 4:** Biological relevance of top-ranked predictive features identified in Dataset-2 and their associated pathways.

**Feature**	**Functional Type**	**Associated Biological Pathway / Mechanism**	**Representative Gene**	**Biological Interpretation**
**phyloP100way/ phyloP470way**	Evolutionary conservation score	Homologous recombination repair, DNA double-strand break response	*BRCA1, BRCA2, RAD51*	Highly conserved residues mark essential domains; variants here often impair HR-mediated repair.
**MetaRNN_score**	Ensemble deep-learning pathogenicity predictor	DNA damage signalling and checkpoint control	*TP53, ATM*	Integrates deleteriousness metrics capturing p53- and ATM-mediated repair disruption.
**MetaLR / MetaSVM**	Meta-predictors combining functional and conservation data	Protein stability and transcriptional regulation	*CHEK2, PALB2*	Reflect cumulative loss-of-function effects in DNA-repair co-factors.
**GERP+ +_RS**	Evolutionary constraint	Genome integrity maintenance, chromatin remodelling	*BRCA1, XRCC2*	Identifies residues under strong purifying selection essential for genomic stability.
**SiPhy_29way_logOdds**	Multi-species conservation	Replication fork protection and DNA synthesis fidelity	*RAD51C, NBN*	Indicates intolerance to variation in replication fork components.
**PrimateAI_score**	Neural network predictor using primate divergence	Estrogen-receptor signalling, transcriptional control	*ESR1, FOXA1*	Suggests selective pressure in hormone-responsive regulatory elements.
**DEOGEN2_score**	Context-aware deleteriousness predictor	Signal transduction and apoptosis	*PTEN, AKT1*	Detects physicochemical disruptions driving aberrant survival signaling.
**MPC_score**	Missense constraint z-score	Protein structure–function coupling	*BRCA2, CDH1*	High MPC denotes intolerance to structural perturbations in tumor-suppressor domains.
**SpliceAI_score**	Deep-learning splicing predictor	RNA splicing and exon-skipping regulation	*BRCA1, MSH2*	Highlights variants altering splice junctions and transcript integrity.

**Fig. 7:**
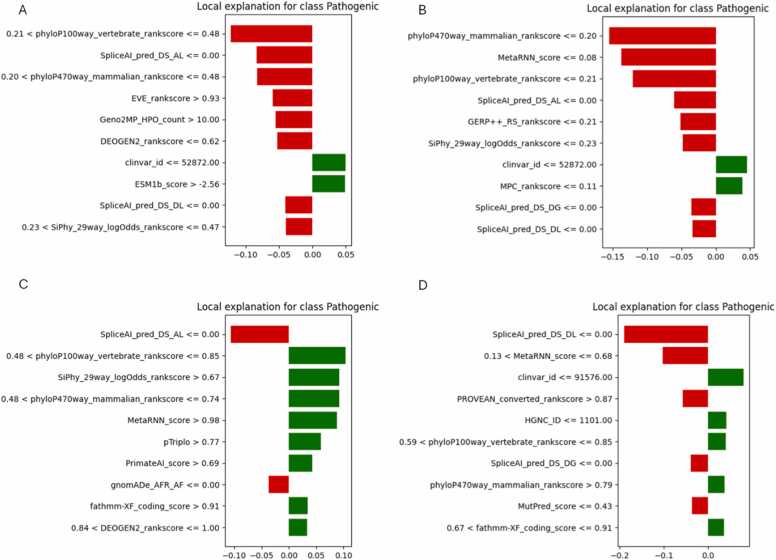
LIME explanations for Dataset-2 using H2O AutoML. (A) FP, (B) TN, (C) TP, (D) FN.

**Fig. 8:**
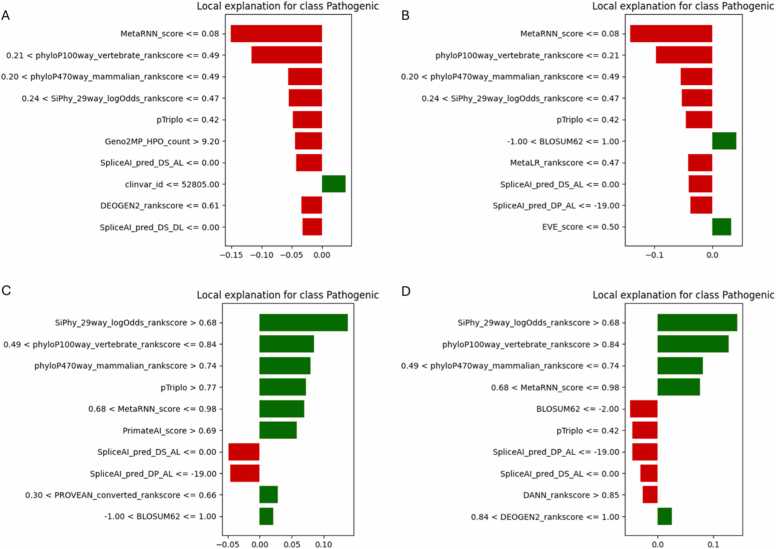
LIME explanations for Dataset-2 using MLJAR AutoML. (A) FP, (B) TN, (C) TP, (D) FN.

**Fig. 9:**
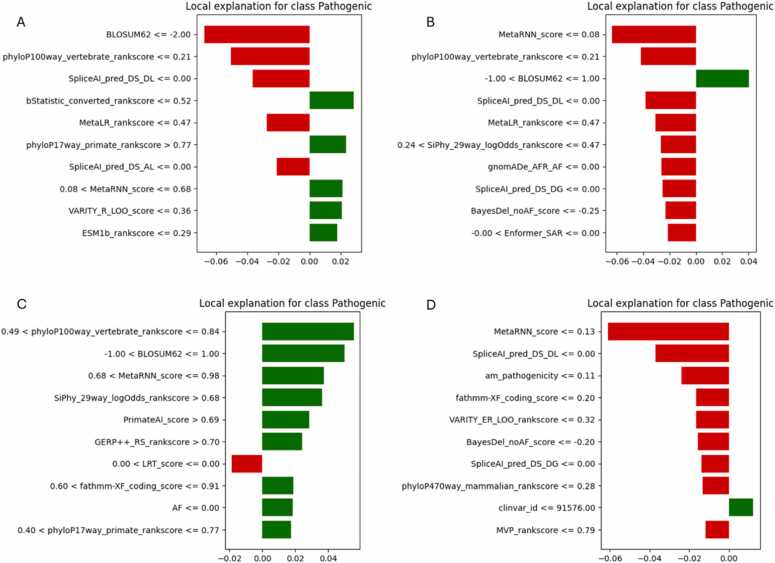
LIME explanations for Dataset-2 using TPOT AutoML. (A) FP, (B) TN, (C) TP, (D) FN.

To provide case-specific interpretability, LIME (Local Interpretable Model-Agnostic Explanations) was applied to one example each of true positive (TP), true negative (TN), false positive (FP), and false negative (FN) predictions for Dataset-2 across all three AutoML frameworks. This helped illuminate how individual features influenced specific predictions and whether they aligned with global feature importance patterns. Fig. 7 presents the LIME explanations for H2O AutoML. The FP explanation shown in Fig. 7A was driven by mid-to-low conservation scores (phyloP100way, SpliceAI, EVE), falsely suggesting pathogenicity. The TN case in Fig. 7B showed a dominant suppressive influence from conservation and splicing scores, successfully supporting a benign label. The TP prediction in Fig. 7C relied on high values from MetaRNN, pTriplo, and PrimateAI, indicating robust use of pathogenic indicators. Conversely, the FN case in Fig. 7D reflected a mixture of weak feature thresholds, such as borderline MetaRNN and DEOGEN2, potentially leading to misclassification. Fig. 8 displays MLJAR’s local explanations. Similar to H2O, the FP case in Fig. 8A showed suppression by ensemble scores like MetaRNN, despite supportive signals from DEOGEN2 and SpliceAI. The TN in Fig. 8B and TP cases in Fig. 8C clearly utilized strong, directional influences from phyloP and MetaLR, validating MLJAR’s interpretability strength. The FN case Fig. 8D illustrated that features like BLOSUM62, although normally predictive, lacked sufficient magnitude to guide a correct outcome. Fig. 9 illustrates TPOT’s local explanations. The FP example in Fig. 9A over-relied on BLOSUM62 and SpliceAI, whereas the TN in Fig. 9B used ensemble scores like MetaLR and VARITY effectively. The TP case in Fig. 9C showed high-confidence decisions stemming from MetaRNN, SiPhy, and PrimateAI. The FN in Fig. 9D reflected weak or misaligned feature thresholds-such as borderline MetaRNN and underweighted fathmm-indicating TPOT’s greater sensitivity to feature value ranges in edge cases. Overall, these LIME analyses confirm that high-confidence predictions often aligned with global SHAP rankings, while misclassifications frequently involved borderline thresholds or conflicting signals. MLJAR explanations were the most interpretable, while TPOT exhibited broader variability.

**Fig. 10:**
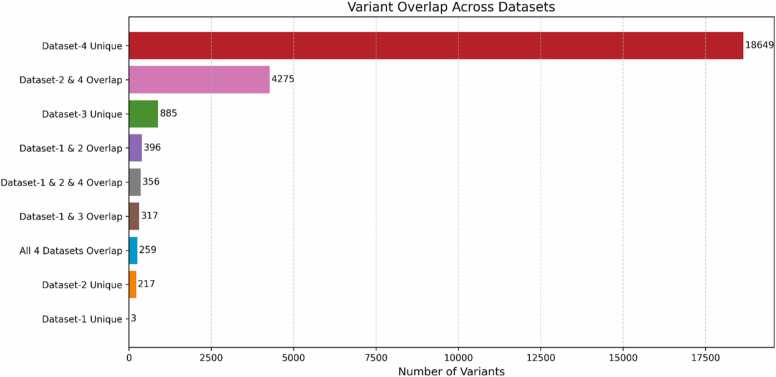
Overlapping variants across datasets.

The composition of the final pipelines varied notably across AutoML frameworks, reflecting their distinct optimization strategies and how they adapt to dataset characteristics. TPOT, which evolves pipelines through genetic programming, produced diverse model architectures tailored to each dataset. For example, Dataset-1 yielded a more complex pipeline involving a Stochastic Gradient Descent classifier combined with a Random Forest, likely to mitigate its smaller size and limited specificity. In contrast, Dataset-2 required only a fine-tuned Random Forest classifier, indicating that its curated content provided a strong enough signal for simpler, yet highly effective models. H2O AutoML exhibited greater consistency, with Distributed Random Forest (DRF) and Stacked Ensemble models emerging as top performers across all datasets. This reflects H2O's emphasis on scalability and ensemble robustness, which suits heterogeneous or high-dimensional datasets. MLJAR AutoML, leveraging its “Compete” mode, automatically benchmarked multiple algorithms-including LightGBM, XGBoost, and CatBoost-and selected the best-performing pipeline based on internal validation, streamlining model development and interpretation. These framework-specific strategies interacted uniquely with each dataset’s composition, influencing model complexity, training efficiency, and generalization ability. Complementing these pipeline-level findings, Fig. 10 illustrates the overlap of genetic variants across the four datasets. Notably, Dataset-2 shares a large number of variants with the more extensive Dataset-4 (4275 variants), suggesting that it captures a biologically rich subset from broader pan-cancer data. Simultaneously, it maintains 217 unique variants-highlighting its disease specificity. Only 259 variants are shared across all datasets, confirming minimal redundancy and reducing the likelihood of performance inflation due to variant duplication. This balanced structure-comprising shared, disease-relevant variants and uniquely curated entries-explains Dataset-2’s superior predictive performance. The variant overlap analysis shown in Fig. 10, revealed a small degree of redundancy across datasets, indicating limited potential for data leakage or performance inflation. Although an explicit exclusion test was not conducted, the stratified splitting strategy and gene-level balancing during preprocessing minimized the influence of shared variants on model training. Future work will include sensitivity analyses excluding overlapping entries to empirically confirm their negligible impact on model generalizability. The dataset’s ability to consistently support high-performing models across TPOT, H2O, and MLJAR demonstrates the importance of dataset composition in pathogenicity prediction. Altogether, these findings position Dataset-2 as the most effective resource for BC variant classification. The convergence of high accuracy, biological interpretability, and pipeline reproducibility across frameworks underscores the value of careful dataset curation. Additionally, the complementary strengths of TPOT, H2O, and MLJAR offer a robust ensemble of AutoML tools for accelerating future clinical applications in precision oncology.

## 4 Discussion

This study comprehensively benchmarked three AutoML frameworks-TPOT, H2O, and MLJAR-across four curated BC variant datasets to evaluate how dataset composition influences pathogenicity prediction performance. By integrating statistical preprocessing, seed-based evaluation and model interpretability, our findings address the pivotal but underexplored role of dataset design in genomic predictive modelling.

Our results demonstrate that Dataset-2, composed of BC-specific variants sourced from cancer-focused repositories like COSMIC, BRCAExchange, and cBioPortal, consistently achieved the highest performance across all frameworks. This dataset not only outperformed broader pan-cancer and general clinical datasets (Datasets 1, 3, and 4), but also showed superior AUC, precision, and calibration under diverse modelling strategies. These findings strongly reinforce the value of disease-specific and high-quality datasets in enabling precise and clinically meaningful variant classification.

Crucially, the seed-based performance stability analysis confirmed Dataset-2's robustness, while the SHAP and LIME interpretability results revealed consistent biological signals across frameworks. Conservation-based scores (e.g., phyloP, GERP++) and ensemble predictors (MetaRNN, DEOGEN2, SiPhy) were the top contributors to predictions, aligning with known biological mechanisms in pathogenicity. TPOT demonstrated flexibility through diverse pipeline composition, H2O excelled with scalable ensembles, and MLJAR balanced interpretability and accuracy-offering complementary strengths for clinical integration.

Additionally, the variant overlap analysis supported the strategic value of Dataset-2. It retains substantial overlap with broader datasets (e.g., 4275 shared variants with Dataset-4) while preserving unique content (217 variants), positioning it as both representative and distinct. This combination likely underpins its consistent model performance, minimizing redundancy while maximizing informative signal.

From a translational perspective, these findings hold significant implications for clinical genomics and precision oncology. Dataset-2 can serve as a strong foundation for developing clinically deployable models, guiding diagnostic pipelines, and informing variant prioritization. Moreover, the interpretability insights from LIME and SHAP offer a transparent basis for clinical validation, enhancing trust in AI-assisted decision-making. The identification of Dataset-2 as optimal has direct translational implications. Clinically, such a dataset could serve as the foundation for disease-specific variant interpretation pipelines in medical diagnostic laboratories. Integrating Dataset-2 into existing clinical genomics workflows, such as *BRCA1/BRCA2* variant reclassification, would allow more precise filtering of VUSs and support clinical decision systems following ACMG/AMP guidelines. Variant-level predictions can be directly incorporated into clinical pipelines for VUS reclassification or tumor-sequencing workflows. By exporting calibrated probabilities and SHAP-based feature summaries, our framework can augment ACMG/AMP-guided variant interpretation in diagnostic laboratories. The prominence of conservation-based and ensemble predictors reflects biological mechanisms underlying homologous-recombination repair and DNA-damage response pathways, central to BRCA-related tumorigenesis. Unexpectedly high contributions from deep-learning predictors like Enformer_SAR suggest potential regulatory or splicing effects beyond canonical coding regions, warranting further exploration. The AutoML framework aligns conceptually with Clinical Decision Support Systems (CDSS) as defined under FDA and EMA guidance for AI-driven medical devices. Future development will emphasize transparency, auditability, and model explainability in accordance with Good Machine Learning Practice (GMLP) principles, facilitating regulatory readiness and potential integration within accredited clinical genomics platforms.

Systems-level interpretation of predictive features revealed that the top-ranked variables identified by AutoML frameworks correspond to well-established breast-cancer pathways. As summarized in Table 4, conservation-based metrics such as phyloP and GERP+ + mapped to homologous-recombination and DNA-damage-repair genes (*BRCA1/2*, *RAD51*), while ensemble predictors like MetaRNN and MetaLR captured disruptions in checkpoint signaling (*TP53*, *ATM*, *CHEK2*). Additional features such as PrimateAI and SpliceAI reflect regulatory and splicing alterations linked to estrogen-receptor and mRNA-processing pathways. This concordance between computational feature importance and known oncogenic mechanisms reinforces the biological validity of Dataset-2 and enhances interpretability for translational applications. Breast cancer represents a dynamic, feedback-regulated system characterized by redundancy and robustness in DNA-repair and signaling pathways. Modeling this complexity through network-based AutoML or graph neural networks could enhance our framework’s ability to capture emergent behaviors beyond single-variant effects.

Comparative runtime analysis under identical Google Colab GPU settings indicated average optimization times of approximately 48 min per dataset for MLJAR AutoML, 55 min for H2O AutoML, and 90 min for TPOT. H2O demonstrated superior scalability for larger genomic inputs, while MLJAR achieved the fastest prototyping, emphasizing the importance of computational efficiency in clinical deployment.

Despite these advances, several limitations merit discussion. First, the current study did not include an independent external test dataset, which limits our ability to evaluate generalizability beyond the datasets analysed. Future work should therefore incorporate externally validated cohorts, ideally derived from real-world patient data, to confirm predictive robustness in clinical settings. Second, potential inconsistencies in variant pathogenicity labels across public databases may have influenced model learning and evaluation. Although we mitigated this risk through dataset harmonization and redundancy removal, inherent discrepancies between ClinVar, HGMD, and other repositories remain a recognized challenge in variant curation, but we tried to overcome this by annotating all variants and using a common ID through VEP. Third, while the AutoML pipelines demonstrated reproducible results across random seeds, their adaptability to diverse variant contexts-particularly somatic versus germline variants-requires further benchmarking to ensure reliable cross-context application. Additionally, although KNN imputation proved effective, exploring biologically informed imputation strategies such as graph-based or pathway-aware models could further enhance data fidelity. Although our framework models variant-level predictions, future extensions will integrate systems biology perspectives by incorporating gene–gene interaction networks (STRING, BioGRID), and cross-omics correlations to model variants within biological context. Finally, while MLJAR integration improved interpretability, assessing its translation into real-world variant annotation workflows will be critical. All reported results were obtained using 5-fold cross-validation, with 95 % confidence intervals estimated via 1,000-sample bootstrapping, confirming statistical robustness and metric stability.

This study distinguishes itself from previous AutoML-based cancer prediction efforts by centring on dataset benchmarking, rather than solely on model comparison. By doing so, it provides a critical methodological contribution to the design of disease-specific datasets for predictive genomics, with immediate applicability to clinical workflows and translational research. All analyses used de-identified, publicly available data. For clinical translation, future deployment of AutoML-based pipelines will adhere to HIPAA and GDPR standards, integrating privacy-preserving techniques such as federated learning and secure data enclaves.

## 5 Conclusions

This study underscores the central role of dataset design in genomic pathogenicity prediction and demonstrates how AutoML frameworks-TPOT, H2O, and MLJAR-can be leveraged to systematically evaluate dataset performance. Among the four curated datasets, Dataset-2, a BC-specific variant set derived from cancer-focused sources, emerged as the most effective for training high-performing and interpretable models. Its balance of specificity, data volume, and feature diversity consistently translated into superior metrics across all frameworks. The interpretability analyses revealed strong biological consistency, with features such as phyloP, MetaRNN, and DEOGEN2 driving predictions across models. TPOT’s genetic programming offered structural flexibility, H2O delivered robust ensemble models, and MLJAR provided streamlined performance with enhanced transparency-collectively showcasing the practical complementarity of AutoML tools in biomedical contexts. Moving forward, the benchmarking pipeline established here can be extended to other disease areas, supporting the development of precision medicine solutions. Future work will explore hybrid AutoML pipelines, real-time model deployment in clinical settings, integration with variant annotation platforms and external validation on a unique dataset. From an ethical perspective for clinical translation, AutoML-based predictors can be deployed within secure decision-support environments compliant with HIPAA and GDPR standards. Integrating privacy-preserving techniques such as federated learning will enable distributed model training across hospital networks without exposing sensitive genomic data. By addressing a critical gap in dataset benchmarking for pathogenicity prediction, this study lays the groundwork for next-generation, disease-specific variant classification systems, enabling earlier diagnosis, better treatment decisions, and improved patient outcomes in BC care and beyond. To improve accessibility and adoption, a prototype web interface and PathoPred was developed. The platform enables users to upload variant data (csv or tabular format), perform inference using pre-trained models derived from Dataset-2, and visualize interpretable SHAP-based variant prioritization results.

## CRediT authorship contribution statement

**Bassam R. Ali:** Writing – review & editing, Visualization, Supervision, Conceptualization. **Mohd Saberi Mohamad:** Writing – review & editing, Supervision, Conceptualization. **Rahaf M. Ahmad:** Writing – original draft, Visualization, Validation, Methodology, Data curation, Conceptualization. **Noura AlDhaheri:** Writing – review & editing, Supervision, Conceptualization.

## Consent for publication

Not applicable.

## Ethics approval and consent to participate

Not applicable.

## Declaration of Generative AI and AI-assisted technologies in the writing process

During the preparation of this work the authors used ChatGPT (OpenAI GPT-5) under direct supervision for language refinement and formatting. After using this tool/service, the author(s) reviewed and edited the content as needed and takes full responsibility for the content of the published article.

## Funding

This work was supported by the United Arab Emirates University through the Strategic Research Program (Grant #12R111). Rahaf M. Ahmad is supported by a PhD fellowship from the United Arab Emirates University. The funding source had no role in study design, data collection and analysis, manuscript preparation, or the decision to submit the article for publication. All authors have read and approved the final version of the manuscript.

## Declaration of Competing Interest

The authors declare that they have no conflict of interest, competing financial interests or personal relationships that could have appeared to influence the work reported in this paper.

## Data Availability

The datasets generated and analysed during the current study are available in the COSMIC (https://cancer.sanger.ac.uk/cosmic), CBioPortal (https://www.cbioportal.org/datasets), BRCAExchange (https://brcaexchange.org/), TCGA (https://www.cancer.gov/ccg/research/genome-sequencing/tcga), ClinVar (https://www.ncbi.nlm.nih.gov/clinvar/) and HGMD Professional 2024.3 (https://www.hgmd.cf.ac.uk/). The final data structure used in this research is available on the GitHub repository. The final dataset is not publicly available due to the inclusion of data from the HGMD Professional version 2024.3 which is subscription based. A larger sample of the dataset can be available from the authors on reasonable request. The code used to generate the results is available on https://github.com/rahafahmad89/AutoML_Pathogenicity_Prediction. A publicly accessible demonstration web application, PathoPred, was developed to facilitate community use and feedback. The web tool is available at https://huggingface.co/spaces/rahafahmad89/PathoPred.
